# Supplementary material for: The blue carbon of southern southwest Atlantic salt marshes and their biotic and abiotic drivers
Source: Nat Commun. 2023 Dec 22;14:8500. doi: 10.1038/s41467-023-44196-w (PMC10746709; doi:10.1038/s41467-023-44196-w)
Supplement: Supplementary file 1 — Supplementary Information [file 41467_2023_44196_MOESM1_ESM.pdf]

Supplementary Information

The Blue Carbon of southern southwest Atlantic salt marshes and their biotic and abiotic drivers

Paulina Martinetto[1*], Juan Alberti[1], María Eugenia Becherucci[1], Just Cebrian[2,†], Oscar Iribarne[1], Núria Marbà[3], Diana Montemayor[1], Eric Sparks[4,5], Raymond Ward[6,7,8]

1: Laboratorio de Ecología, Instituto de Investigaciones Marinas y Costeras (IIMyC, UNMdP-CONICET), Juan B Justo 2550, Mar del Plata (7600), Argentina

2: Northern Gulf Institute, Mississippi State University, NOAA NCEI, 1021 Balch Blvd Stennis Space Center, MS 39529, USA

3: Global Change Research Group, IMEDEA (CSIC-UIB), Institut Mediterrani d'Estudis Avançats, Miquel Marquès 21, 07190 Esporles (Illes Balears), Spain

4: Coastal Research and Extension Center, Mississippi State University, 1815 Popp's Ferry Rd., Biloxi, MS 39532, USA

5: Mississippi-Alabama Sea Grant Consortium, 703 East Beach Drive, Ocean Springs, MS 39564, USA

6: School of Geography, Queen Mary University of London, Mile End Rd, Bethnal Green, London E1 4NS, United Kingdom.

7: Institute of Agriculture and Environmental Sciences, Estonia University of Life Sciences, Kreutzwaldi 5, EE-51014 Tartu, Estonia.

8: Colégio de Estudos Avançados, Universidade Federal do Ceará, Campus do Pici - CEP 60455-760 - Fortaleza – CE, Brasil

*Corresponding author: P. Martinetto pmartin@mdp.edu.ar

†: JC Current address: "Vesta, PBC", 584 Castro St, #2054, San Francisco, CA 94114-2512, USA

Supplementary Information Fig. 1: Activity/depth profiles for ^{210}Pb excess (blue diamonds) and ^{214}Pb (orange squares) as a proxy for ^{210}Pb supported activity for the upper and lower salt marshes studied.

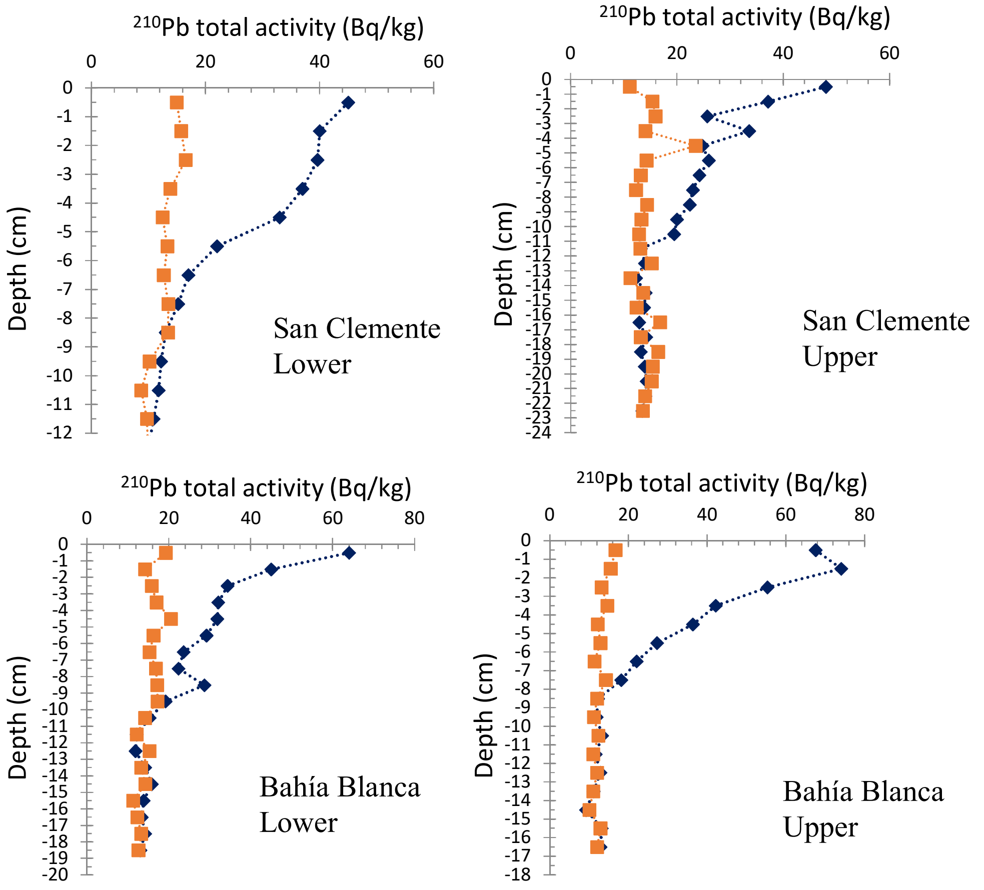

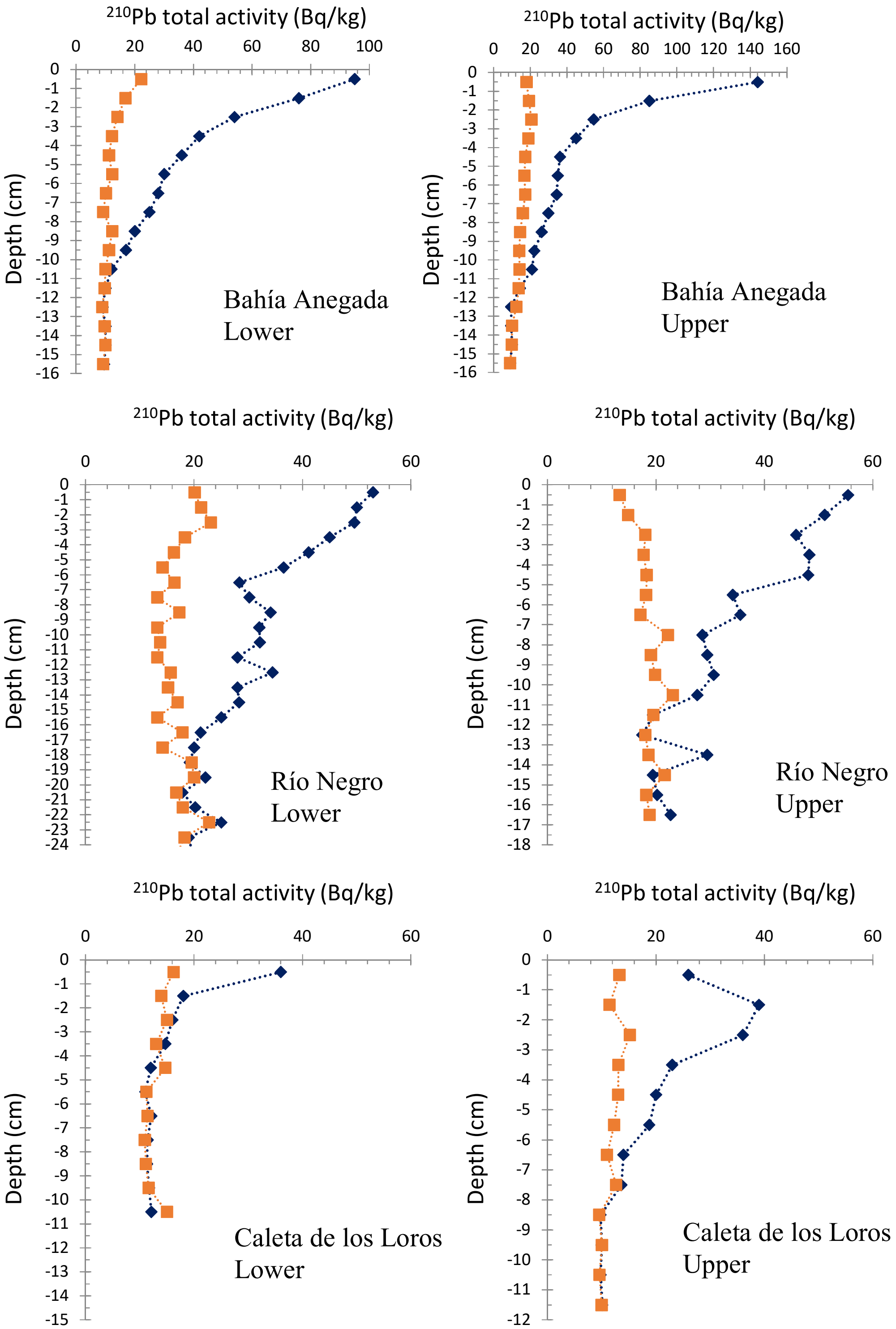

^{210}Pb total activity (Bq/kg)
Depth (cm)
Bahía Anegada
Lower
^{210}Pb total activity (Bq/kg)
Depth (cm)
Bahía Anegada
Upper
^{210}Pb total activity (Bq/kg)
Depth (cm)
Río Negro
Lower
^{210}Pb total activity (Bq/kg)
Depth (cm)
Río Negro
Upper
^{210}Pb total activity (Bq/kg)
Depth (cm)
Caleta de los Loros
Lower
^{210}Pb total activity (Bq/kg)
Depth (cm)
Caleta de los Loros
Upper

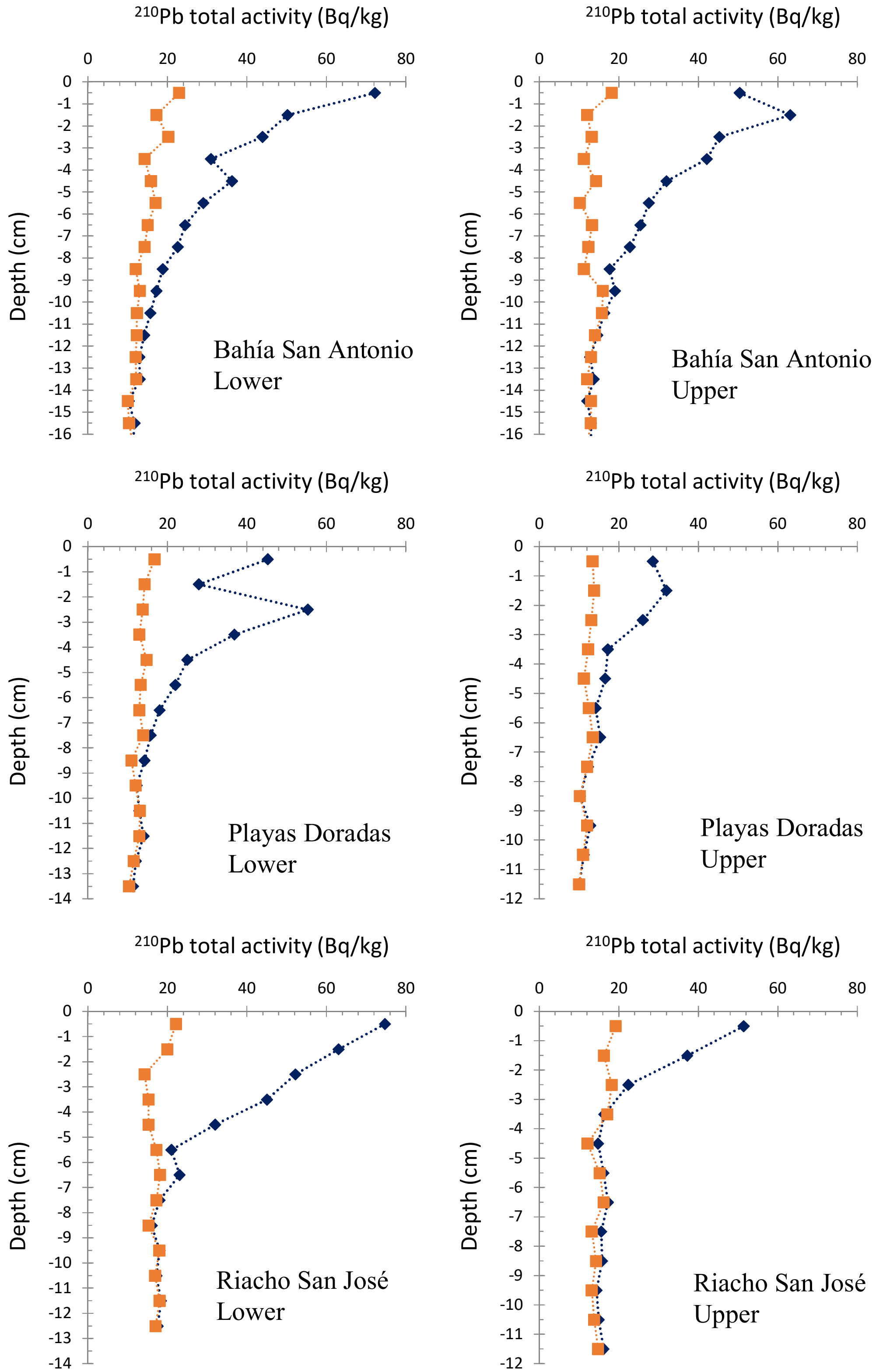

^{210}Pb total activity (Bq/kg)
0 20 40 60 80
Depth (cm)
Bahía San Antonio Lower
^{210}Pb total activity (Bq/kg)
Depth (cm)
Bahía San Antonio Upper
^{210}Pb total activity (Bq/kg)
Depth (cm)
Playas Doradas Lower
^{210}Pb total activity (Bq/kg)
Depth (cm)
Playas Doradas Upper
^{210}Pb total activity (Bq/kg)
Depth (cm)
Riacho San José Lower
^{210}Pb total activity (Bq/kg)
Depth (cm)
Riacho San José Upper

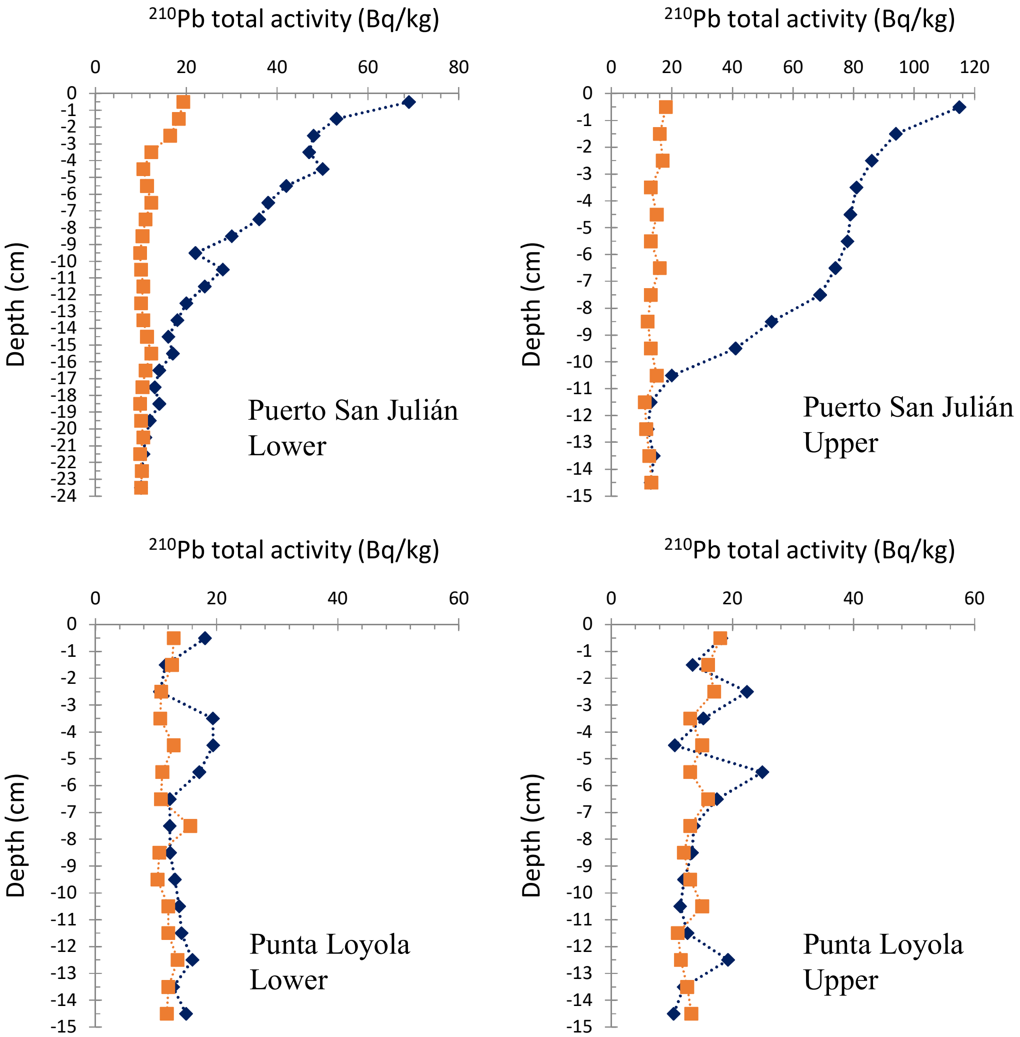

^{210}Pb total activity (Bq/kg)
Depth (cm)
Puerto San Julián Lower
^{210}Pb total activity (Bq/kg)
Depth (cm)
Puerto San Julián Upper
^{210}Pb total activity (Bq/kg)
Depth (cm)
Punta Loyola Lower
^{210}Pb total activity (Bq/kg)
Depth (cm)
Punta Loyola Upper

Supplementary Information Table 1. Mean (SE) of isotopic nitrogen and carbon values (‰) of salt marsh plant species and surficial sediments (2cm depth) collected at the eleven study sites. N indicates the number of samples analysed; it is indicated also when different sample numbers were analysed for Nitrogen (N) and Carbon (C). NM means no material found in the sampling units.

Site	Plant species	Plant part	N	δ^{15}N (‰)	δ^{13}C (‰)
Bahía Samborombón	*S. alterniflora*	Green leaf	5	9.8 (0.7)	-12.85 (0.2)
		Senescent leaf	4	6.7 (0.6)	-12.3 (0.55)
		Roots	5	5.95 (0.7)	-13.4 (0.9)
		Sediment	5	9.9 (0.3)	-19.3 (0.3)
	S. densiflora	Green leaf	5 N, 4C	2.7 (0.03)	-12.8 (0.1)
		Senescent leaf	3 N, 5C	0.8 (0.4)	-12.8 (0.03)
		Roots	5	2.7 (0.3)	-12.1 (0.5)
		Sediment	5	7.3 (1.0)	-17.2 (0.2)
	Salicornia sp.	Green leaf	5	7.9 (0.3)	-27.5 (0.1)
		Senescent leaf	4	6.8 (0.3)	-27.3 (0.2)
		Roots	5	6.7 (0.4)	-20.0 (3.2)
		Sediment	5	9.1 (0.8)	-22.8 (0.3)
Mar Chiquita	*S. densiflora*	Green leaf	5	6.6 (0.6)	-13.25 (0.1)
		Senescent leaf	5	3.7 (0.6)	-12.9 (0.1)

		Roots	5	4.6 (0.6)	-15.8 (2.4)
		Sediment	4	7.7 (1.2)	-18.7 (0.4)
Bahía Blanca	*S. alterniflora*	Green leaf	5	11.2 (0.2)	-13.8 (0.05)
		Senescent leaf	5	10.7 (0.7)	-13.1 (0.1)
		Roots	5	10.2 (0.6)	-13.7 (0.1)
		Sediment	5	10.8 (0.4)	-18.4 (0.4)
	S. densiflora	Green leaf	5	11.2 (1.0)	-13.3 (0.1)
		Senescent leaf	5	10.4 (0.3)	-13.05 (0.2)
		Roots	5	10.6 (0.4)	-14.4 (0.9)
		Sediment	5	11.1 (0.2)	-18.4 (0.5)
	Salicornia sp.	Green leaf	5	9.2 (0.2)	-26.0 (0.3)
		Senescent leaf	2	10.1 (1.7)	-25.8 (0.5)
		Roots	5	6.6 (0.5)	-26.2 (0.4)
		Sediment	5	9.3 (0.1)	-18.2 (0.4)
Bahía Anegada	*S. alterniflora*	Green leaf	5	12.8 (0.7)	-13.1 (0.05)
		Senescent leaf	5	12.6 (1.6)	-14.0 (0.5)
		Roots	5	11.6 (1.65)	-12.5 (0.5)
		Sediment	5	9.5 (0.65)	-20.2 (0.6)
	Salicornia sp.	Green leaf	1	12.1	-26.7
		Senescent leaf	1	1.65	-25.5

		Roots	1	3.5	-26.8
		Sediment	4	6.4 (0.2)	-19.0 (0.7)
Río Negro	*S. alterniflora*	Green leaf	3	9.2 (0.6)	-17.0 (4.05)
		Senescent leaf	3	8.7 (0.2)	-13.35 (0.2)
		Roots	2	4.4 (0.15)	-13.6 (0.1)
		Sediment	3	10.05 (0.5)	-21.0 (1.7)
	S. densiflora	Green leaf	2	4.7 (0.7)	-13.4 (0.1)
		Senescent leaf	2	2.6 (0.4)	-13.5 (0.15)
		Roots	2	4.8 (0.1)	-15.2 (0.6)
		Sediment	3	8.0 (0.1)	-18.7 (0.8)
Caleta Los Loros	*S. alterniflora*	Green leaf	5	9.0 (0.2)	-13.0 (0.1)
		Senescent leaf	5	7.6 (0.6)	-15.6 (2.0)
		Roots	5	5.85 (0.2)	-13.5 (0.04)
		Sediment	5	5.4 (0.4)	-19.1 (0.6)
	Salicornia sp.	Green leaf	3	6.3 (0.4)	-25.05 (0.2)
		Senescent leaf	1	4.8	-24.6
		Roots	3	6.6 (1.9)	-24.7 (0.75)
		Sediment	3	8.1 (0.55)	-14.6 (0.6)
Bahía San Antonio	*S. alterniflora*	Green leaf	5	6.6 (0.8)	-13.2 (0.2)

		Senescent leaf	5	5.0 (0.3)	-13.2 (0.1)
		Roots	4	5.8 (0.4)	-13.55 (0.3)
		Sediment	5	10.8 (0.5)	-18.6 (1.4)
	Salicornia sp.	Green leaf	5	8.2 (0.6)	-26.7 (0.5)
		Senescent leaf	3	6.4 (1.7)	-20.9 (3.9)
		Roots	5	5.15 (0.4)	-26.6 (0.4)
		Sediment	5	9.85 (0.6)	-18.4 (0.3)
Playas Doradas	*S. alterniflora*	Green leaf	5	10.5 (0.2)	-25.8 (0.3)
		Senescent leaf	NM		
		Roots	5	5.7 (1.1)	-23.2 (2.6)
		Sediment	5	11.5 (0.8)	-22.2 (1.2)
Riacho San José	*S. alterniflora*	Green leaf	5	10.8 (0.1)	-13.1 (0.1)
		Senescent leaf	5	8.9 (0.4)	-13.1 (0.05)
		Roots	5	6.1 (0.2)	-17.2 (1.0)
		Sediment	5	7.55 (0.55)	-20.2 (0.6)
	densiflora	Green leaf	4	8.4 (0.9)	-13.2 (0.1)
		Senescent leaf	5	7.8 (1.1)	-12.4 (0.2)
		Roots	3	8.0 (0.6)	-13.8 (0.3)
		Sediment	3	10.6 (0.4)	-17.9 (0.4)
Puerto San	*Salicornia sp.*	Green leaf	5	10.0 (0.4)	-24.7 (0.2)

Julián		Senescent leaf	1	7.2	-25.0
		Roots	5	8.8 (0.4)	-24.3 (0.35)
		Sediment	5	9.7 (0.2)	-21.2 (1.6)
Punta Loyola	*Salicornia sp.*	Green leaf	5	6.9 (0.4)	-26.6 (0.3)
		Senescent leaf	1	10.4	-26.15
		Roots	5	7.9 (0.4)	-23.1 (2.25)
		Sediment	3	9.4 (0.3)	-24.6 (0.5)

Supplementary Information Table 2. Mean, quantiles (50, 2.5 and 97.5%) and SD of proportions of each carbon source for the surficial sediments (2cm depth) of each sampling site and according to plant species dominant of the salt marsh. POM is particulate organic matter. Asterisk (*) indicates the major median source contribution value when differentiate from the other sources. The quantiles 2.5 and 97.5% are Bayesian credible intervals. Dash (-) indicates organic matter source or dominant plant species no present in the site.

	Spartina alterniflora			*Spartina densiflora*			*Salicornia* sp.		
	POM	Plant	Macroalgae	POM	Plant	Macroalgae	POM	Plant	Macroalgae
**Bahía Samborombón**									
Mean	0.80	0.20	-	0.57	0.42	-	0.42	0.58	-
SD	0.09	0.09	-	0.05	0.05	-	0.17	0.17	-
2.5%	0.58	0.05	-	0.46	0.32	-	0.10	0.24	-
50%	0.81*	0.19	-	0.57	0.43	-	0.42	0.58	-
97.5%	0.65	0.42	-	0.68	0.54	-	0.76	0.90	-
**Mar Chiquita**									
Mean	-	-	-	0.80	0.20	-	-	-	-
SD	-	-	-	0.08	0.08	-	-	-	-
2.5%	-	-	-	0.61	0.05	-	-	-	-
50%	-	-	-	0.80*	0.19	-	-	-	-
97.5%	-	-	-	0.95	0.39	-	-	-	-
**Bahía Blanca**									
Mean	0.77	0.23	-	0.75	0.25	-	0.86	0.14	-

SD	0.10	0.10	-	0.14	0.14	-	0.13	0.13	-
2.5%	0.55	0.03	-	0.43	0.03	-	0.52	0.01	-
50%	0.77*	0.23	-	0.77	0.23	-	0.89*	0.10	-
97.5%	0.97	0.45	-	0.97	0.57	-	0.99	0.47	-
**Bahía Anegada**									
Mean	0.91	0.09	ND	-	-	-	0.88	0.12	-
SD	0.08	0.08	ND	-	-	-	0.10	0.10	-
2.5%	0.71	0.01	ND	-	-	-	0.63	0.01	-
50%	0.93*	0.07	ND	-	-	-	0.90*	0.09	-
97.5%	0.99	0.29	ND	-	-	-	0.99	0.37	-
**Bahía San Antonio**									
Mean	0.56	0.24	0.19	-	-	-	0.58	0.18	0.24
SD	0.20	0.13	0.17	-	-	-	0.21	0.09	0.17
2.5%	0.09	0.02	0.01	-	-	-	0.10	0.02	0.01
50%	0.58	0.24	0.15	-	-	-	0.61	0.18	0.21
97.5%	0.89	0.49	0.63	-	-	-	0.90	0.37	0.63
**Río Negro**									
Mean	0.72	0.28	-	0.60	0.40	-	-	-	-
SD	0.18	0.18	-	0.16	0.16	-	-	-	-
2.5%	0.28	0.01	-	0.33	0.06	-	-	-	-
50%	0.74	0.26	-	0.57	0.42	-	-	-	-
97.5%	0.98	0.72	-	0.93	0.67	-	-	-	-
**Caleta Los Loros**									

Mean	0.55	0.45	-	-	-	-	0.74	0.26	-
SD	0.23	0.23	-	-	-	-	0.26	0.26	-
2.5%	0.10	0.04	-	-	-	-	0.05	0.01	-
50%	0.55	0.45	-	-	-	-	0.83	0.17	-
97.5%	0.96	0.90	-	-	-	-	0.99	0.95	-
**Playas Doradas**									
Mean	-	-	-	-	-	-	0.73	0.27	-
SD	-	-	-	-	-	-	0.16	0.16	-
2.5%	-	-	-	-	-	-	0.35	0.03	-
50%	-	-	-	-	-	-	0.76	0.24	-
97.5%	-	-	-	-	-	-	0.97	0.65	-
**Riacho San José**									
Mean	0.58	0.42	-	0.59	0.41	-	-	-	-
SD	0.22	0.22	-	0.06	0.06	-	-	-	-
2.5%	0.06	0.11	-	0.45	0.30	-	-	-	-
50%	0.61	0.39	-	0.60	0.40	-	-	-	-
97.5%	0.89	0.93	-	0.70	0.55	-	-	-	-
**Puerto San Julián**									
Mean	-	-	-	-	-	-	0.21	0.56	0.22
SD	-	-	-	-	-	-	0.18	0.15	0.14
2.5%	-	-	-	-	-	-	0.01	0.21	0.01
50%	-	-	-	-	-	-	0.17	0.58	0.22
97.5%	-	-	-	-	-	-	0.65	0.84	0.50

**Punta Loyola**									
Mean	-	-	-	-	-	-	0.41	0.59	-
SD	-	-	-	-	-	-	0.18	0.18	-
2.5%	-	-	-	-	-	-	0.07	0.24	-
50%	-	-	-	-	-	-	0.40	0.59	-
97.5%	-	-	-	-	-	-	0.76	0.93	-

Supplementary Information Table 3. Carbon and nitrogen isotopic signatures of organic matter allochthonous sources from the literature. POM: particulate organic matter.

**Source**	**Site**	**$\delta^{15}N$ (‰) mean (SE)**	**$\delta^{13}C$ (‰) mean (SE)**	**N**	**Reference**
POM	Rio de la Plata estuary (35°S – 56°30'W)	12.5 (3.64)	-20.5 (0.8)	5	1
POM	Rio de la Plata estuary (36°S – 55°30'W)	6.3 (1.9)	-20.1 (1.75)	5	1
Phytoplankton	Golfo Nuevo coast (42°50'S-64°2'W)	13.4 (0.6)	-21.0 (0.1)	2	2
Condium vermilara	Golfo Nuevo coast (42° 47' S, 65° 06' W)	11.6 (0.6)	-14.9 (1.3)	5	2
Undaria pinnatifida	Golfo Nuevo coast (42° 47' S, 65° 06' W)	10.4 (0.5)	-19.1 (1.2)	5	2
Ulva sp.	San Antonio Oeste tidal channels (40°43'S, 64°57'W)	13.6 (3.9)	-18.5 (1.4)	18	3
Ulva lactuca	San Antonio Oeste tidal channels (40°43'S, 64°57'W)	14.5 (2.0)	-13.7 (2.75)	43	3
Microphytobenthos	San Antonio Oeste tidal channels (40°43'S, 64°57'W)	10.2 (0.6)	-14.9 (3.3)	6	3
POM	San Antonio Oeste tidal channels (40°43'S, 64°57'W)	12.7 (1.9)	-18.3 (1.8)	11	3
Ulva sp.	San Antonio Oeste tidal channels (40°72'S, 64°57'W)	9.85 (2.7)	-15.4 (2.75)	17	3
Ulva lactuca	San Antonio Oeste tidal channels	11.5 (4.6)	-12.7 (2.3)	9	3

	(40°72'S, 64°57'W)				
Microphytobenthos	San Antonio Oeste tidal channels (40°72'S, 64°57'W)	9.6 (1.1)	-15.9 (2.0)	6	3
POM	San Antonio Oeste tidal channels (40°72'S, 64°57'W)	10.75 (0.65)	-16.6 (1.9)	8	3
POM	Mar Chiquita (37º 43'S, 57º 23'W)	8.7 (0.4)	-20.4 (0.4)		4

Supplementary Information Table 4. Summary of potential links between dependent and predictive variables incorporated in the SEM analysis and the rationale behind the potential link. Links predicted as positive are indicated with a blue dot, negative with red and neutral with black. OC: organic carbon, OM: organic matter, DOC: dissolved organic carbon, SAR: sediment accretion rate, C:N: carbon and nitrogen ratio. Interpretations for mean grain size are based on the So (sorting coefficient, with larger numbers for larger grains).

**Dependent variable**	**Predicted variables**	**Rationale behind the links**	**References**
OC stocks	● Freshwater input	Increases the availability of sediment and affects OM, related to the type of salt marsh developed; fluvial dominated salt marshes are related to higher C stocks than marine ones	5
	● Mean temperature	Higher temperatures are related to higher decomposition rates	6
	● Annual precipitation	Increase lateral DOC fluxes and decomposition rates	6
	● Sediment depth	Deeper sediments allow both larger rhizome biomass and a larger depth to integrate OC stocks expressed as mass unit per area.	5

	● Mean grain size	Finer sediments are related to higher C stocks; the opposite for coarse sediments	7, 8
	● Dominant plant species	Plant structure affects retention of sediment and C, different plant species have different C contents	9, 10
	● Mean tide	Higher tides can increase export of OM and erosion	5
	● SAR	Higher SAR can increase the accumulation of OC when available, if OC is not available a higher SAR could only increase the accumulation of sediments and dilutes the OC concentration	11, 12
	● Crab burrows	Burrowing activity can either increase retention of organic C or reduce it by increasing aerobic decomposition and exportation to adjacent systems	13, 14
	● C:N Above ground green tissue	Quality of plant tissue affects decomposition rates	15
	● C:N Above ground senescent tissue	Quality of plant tissue affects decomposition rates	15
	● C:N roots	Quality of plant tissue affects decomposition rates	16
	● Above ground green biomass	Plants producing more aboveground biomass can contribute with larger amount of OM	17
	● Above ground senescent biomass	Plants producing more aboveground biomass can contribute with larger amount of OM	17
	● Roots biomass	Root biomass contributes with belowground organic C stocks	17
Crabs	● Mean grain size	Crabs prefer finer sediment to construct their burrows	18
	● Dominant plant species	*Spartina alterniflora* is preferred as food; numbers of burrows change depending on the dominant plant species	19, 20
	● Mean tide	Tide inundation affects the effort to maintain burrows, crab recruitment and physiological and reproductive	21, 22

		processes. It also affects crab migration between tidal flats and salt marshes	
Above ground green biomass	● Crabs	Crabs eat salt marsh plants but also increase the productivity of *Spartina densiflora* by facilitating the association with mycorrhizal fungi	23
	● Freshwater input	Higher freshwater inputs are related to higher productivity	24, 25
	● Mean temperature	Higher temperature can increase productivity but also increase evapotranspiration/desiccation	6
	● Annual precipitation	Lower precipitation is related to decrease in plant growth. Large rainfall periods increase crab herbivory. Large flood periods inhibit *Spartina* spp. growth	26, 20, 27, 28
	● Mean grain size	Finer sediments allow greater plant growth	25
	● Dominant plant species	Different plant species have different biomasses; *Spartina densiflora* usually has > biomass than *S. alterniflora* and *S. alterniflora* > than *Salicornia* sp.	29
Above ground senescent biomass	● Crabs	Crabs increase senescent biomass by facilitating fungal infection of damaged leaves	30, 20
	● Freshwater input	Areas with higher freshwater inputs have lower senescent biomass	31
	● Mean temperature	Extreme temperature can increase senescent biomass	32
	● Annual precipitation	Areas with lower precipitation are more prone to have more senescent biomass	26
	● Mean tide	Larger tides can remove and export senescent tissue	33
	● Dominant plant species	Different plant species have different biomasses; *Spartina densiflora* is more prone to hold > senescent biomass than *S. alterniflora* and *S. alterniflora* > *Salicornia* sp.	34, 29

	● Above ground green biomass	There is usually a positive relationship between green and senescent above ground biomass	32
Roots biomass	● Crabs	Crab burrows interfere with root development	35
	● Freshwater input	Salinity increases root growth and/or growth is relatively higher compared to aerial growth	36, 37
	● Mean temperature	Higher temperatures are related to increased root biomass	32
	● Annual precipitation	Mechanistic effects similar to freshwater input decreasing general salinity	36, 37
	● Mean grain size	Finer sediments are related to higher root biomass than coarser sediments	35, 25
	● Dominant plant species	Different marsh plant species show different belowground biomasses. In this study *Spartina alterniflora* usually has more root biomass than *Spartina densiflora*; *Salicornia* sp. has greater biomass in the southernmost sites	38, this study
	● Sediment depth	Deeper sediments allow larger root biomasses	35

Supplementary References:

1. Botto, F. et al. Origin of resources and trophic pathways in a large SW Atlantic estuary: An evaluation using stable isotopes. *Estuar. Coast. Shelf Sci.* **92**, 70-77 (2011).

2. Drago, M. et al. Ontogenic dietary changes in South American sea lions. *J. Zool.* **279**, 219-318 (2009).

3. Becherucci, M. E. et al. Eutrophication in a semi-desert coastal ecosystem promotes increases in N and C isotopic signatures and changes in primary sources. *Mar. Environ. Res*. **146**, 71-79 (2019).

4. Botto, F. Et al. Impact of burrowing crabs on C and N sources, control, and transformation in sediments and food webs of SW Atlantic estuaries. *Mar. Ecol. Prog. Ser.* **293**,155-164 (2005).

5. Macreadie, P. I. et al. Carbon sequestration by Australian tidal marshes. *Sci. Rep.* **7**, 44071 (2017).

6. Megonigal, J. P. et al. *Coastal wetland responses to warming*. Chapter 11, in: *A Blue Carbon Primer*, L. Windham-Myers, S. Crooks & T. G. Troxler (eds.) CRC Editions, Boca Raton (2018).

7. Kelleway, J. J. et al. Sedimentary factors are key predictors of carbon storage in SE Australian salt marshes. *Ecosystems* **19**, 865–880 (2016).

8. Gorham, C. et al. Soil Carbon Stocks Vary Across Geomorphic Settings in Australian Temperate Tidal Marsh Ecosystems. *Ecosystems* **24**, 319–334 (2021).

9. Kelleway, J. J. et al. Sediment and carbon deposition vary among vegetation assemblages in a coastal salt marsh. *Biogeosciences* **14**, 3763–3779 (2017).

10. Ouyang, X., & Lee, S. Y. Update estimates of carbon accumulation rates in coastal marsh sediments. *Biogeosciences* **11**, 5057–5071 (2014).

11. Lovelock, C.E. et al. Contemporary Rates of Carbon Sequestration Through Vertical Accretion of Sediments in Mangrove Forests and Saltmarshes of South East Queensland, Australia. *Estuar. Coasts* **37**, 763–771 (2014).

12. Gorham, C. et al. Heterogeneous tidal marsh soil organic carbon accumulation among and within temperate estuaries in Australia. *Sci. Tot. Environ*. **787**, 147482 (2021).

13. Botto, F. et al. Ecological importance of passive deposition of organic matter into burrows of the SW Atlantic crab *Chasmagnathus granulatus*. *Mar. Ecol. Prog. Ser.* **312**, 201–210 (2006).

14. Fanjul, E. et al. Effect of crab bioturbation on organic matter processing in South West Atlantic intertidal sediments. *J. Sea. Res.* **94**,194–212 (2014).

15. Valiela, I. et al. Decomposition in salt marsh ecosystems: The phases and major factors affecting disappearance of above-ground organic matter. *J. Exp. Mar. Biol. Ecol*. **89**, 29-54 (1985).

16. Buth, G. J. C. Decomposition of roots of three plant communities in a Dutch salt marsh. *Aquat. Bot*. **29**, 123-138 (1987).

17. Howard, J. et al. (eds.) *Coastal Blue Carbon: Methods for assessing carbon stocks and emissions factors in mangroves, tidal salt marshes, and seagrass meadows*. Conservation International, Intergovernmental Oceanographic Commission of UNESCO, International Union for Conservation of Nature. Arlington, Virginia, USA (2014).

18. Botto, F. & Iribarne, O. Contrasting effects of two burrowing crabs (*Chasmagnathus granulata* and *Uca uruguayensis*) on sediment composition and transport in estuarine environments. *Estuar. Coast. Shelf Sci.* **51**, 141-151 (2000).

19. Martinetto, P. et al. Crab bioturbation and herbivory may account for variability in carbon sequestration and stocks in South West Atlantic salt marshes. *Front. Mar. Sci*. **3**, 122 (2016).

20. Alberti, J. et al. Local and geographic variation in grazing intensity by herbivorous crabs in SW Atlantic saltmarshes. *Mar. Ecol. Prog. Ser.* **349**, 235–243 (2007).

21. Méndez Casariego, A. et al. Habitat shifts and spatial distribution of the intertidal crab *Neohelice* (*Chasmagnathus*) *granulata* Dana*. J. Sea Res.* **66**, 87–94 (2011).

22. Mendez Casariego, A. et al. Increase of organic matter transport between marshes and tidal flats by the burrowing crab *Neohelice (Chasmagnathus) granulata* Dana in SW Atlantic salt marshes. *J. Exp. Mar. Biol. Ecol.* **401**, 110-117 (2011).

23. Daleo, P. et al. Ecosystem engineers activate mycorrhizal mutualism in salt marshes. *Ecol. Lett.* **10,** 902–908 (2007).

24. Isacch, J. P. et al. Distribution of salt marsh plant communities associated with environmental factors along a latitudinal gradient on the south-west Atlantic coast*. J. Biogeography* **33**, 888–900 (2006).

25. Huckle, J. M. et al. Influence of environmental factors on the growth and interactions between salt marsh plants: effects of salinity, sediment and waterlogging. *J. Ecol*. **88**, 492-505 (2000).

26. Pascual, J. et al. Rainfall intensity modulates the interaction between the marsh cordgrass *Spartina densiflora* and the mouse *Akodon azarae*. *Mar. Ecol. Prog. Ser.* **523**, 71-80 (2015).

27. Marangoni, J. C. & Costa, C. S. B. Short-and long-term vegetative propagation of two *Spartina* species on a salt marsh in Southern Brazil. *Estuar. Coast*. **35**, 763–773 (2012).

28. Rocca, C. et al. Flood-stimulated herbivory drives range retraction of a plant ecosystem. *J. Ecol*. **109**, 3541-3554 (2021).

29. Montemayor, D. I. et al. Biomass dynamics of the two dominant SW Atlantic *Spartina* species and its implications on the salt marsh organic matter accumulation/exportation. *Aquat. Bot.* **120**, 201–204 (2015).

30. Daleo, P. et al. Grazer facilitation of fungal infection and the control of plant growth in SW Atlantic saltmarshes. *J. Ecol.* **97**, 781–787 (2009).

31. Hardisky, M. A. et al. Growth response and spectral characteristics of a short *Spartina alterniflora* salt marsh irrigated with freshwater and sewage effluent. *Remote Sens. Environ*. **13**, 57-67 (2983).

32. Cunha, S. R. et al. Production dynamics of *Spartina alterniflora* saltmarshes in the estuary of Patos Lagoon (RS, Brazil): a Simulation model approach. *Brazil. J. Aquat. Sci. Technol. Itajaí* **9**, 75–85 (2005).

33. Gutiérrez, J. L. et al. The contribution of crab burrow excavation to carbon availability in surficial salt-marsh sediments. *Ecosystems* **9**, 647-658 (2006).

34. Montemayor, D. I. et al. Effect of dominant *Spartina* species on salt marsh detritus production in SW Atlantic estuaries. *J. Sea. Res.* **66**, 104–110 (2011).

35. Daleo,P. & Iribarne,O. The burrowing crab *Neohelice granulata* affects the root strategies of the cordgrass *Spartina densiflora* in SW Atlantic salt marshes. *J. Exp. Mar. Biol. Ecol*. **373**, 66–71 (2009).

36. Sutter, L.A. et al. Tidal Freshwater Marsh Plant Responses to Low Level Salinity Increases. *Wetlands* **34**, 167–175 (2014).

37. Alldred, M. et al. Impact of salinity and nutrients on salt marsh stability. *Ecosphere* **8**, e02010 (2017).

38. Tripathee, R. & Schäfer, K.V.R. Above- and Belowground Biomass Allocation in Four Dominant Salt Marsh Species of the Eastern United States. *Wetlands* **35**, 21–30 (2015).
